# The GTPase Arf1 Is a Determinant of Yeast Vps13 Localization to the Golgi Apparatus

**DOI:** 10.3390/ijms222212274

**Published:** 2021-11-12

**Authors:** Damian Kolakowski, Weronika Rzepnikowska, Aneta Kaniak-Golik, Teresa Zoladek, Joanna Kaminska

**Affiliations:** 1 Institute of Biochemistry and Biophysics, Polish Academy of Science, 02-106 Warsaw, Poland; damian.kolakowski@ibb.waw.pl (D.K.); anetak@ibb.waw.pl (A.K.-G.); teresa@ibb.waw.pl (T.Z.); 2 Neuromuscular Unit, Mossakowski Medical Research Institute, Polish Academy of Sciences, 02-106 Warsaw, Poland; wrzepnikowska@imdik.pan.pl

**Keywords:** Arf1, Vps13, VPS13A, Golgi apparatus, mitochondria, PH-like domain, PI(4,5)P_2_, yeast

## Abstract

VPS13 proteins are evolutionarily conserved. Mutations in the four human genes (*VPS13A-D*) encoding VPS13A-D proteins are linked to developmental or neurodegenerative diseases. The relationship between the specific localization of individual VPS13 proteins, their molecular functions, and the pathology of these diseases is unknown. Here we used a yeast model to establish the determinants of Vps13′s interaction with the membranes of Golgi apparatus. We analyzed the different phenotypes of the *arf1-3 arf2*Δ *vps13*∆ strain, with reduced activity of the Arf1 GTPase, the master regulator of Golgi function and entirely devoid of Vps13. Our analysis led us to propose that Vps13 and Arf1 proteins cooperate at the Golgi apparatus. We showed that Vps13 binds to the Arf1 GTPase through its C-terminal Pleckstrin homology (PH)-like domain. This domain also interacts with phosphoinositol 4,5-bisphosphate as it was bound to liposomes enriched with this lipid. The homologous domain of VPS13A exhibited the same behavior. Furthermore, a fusion of the PH-like domain of Vps13 to green fluorescent protein was localized to Golgi structures in an Arf1-dependent manner. These results suggest that the PH-like domains and Arf1 are determinants of the localization of VPS13 proteins to the Golgi apparatus in yeast and humans.

## 1. Introduction

VPS13 proteins belong to an evolutionarily conserved family of proteins. The yeast Vps13 protein was the founding member of this family. Four human members of the family have been identified (*VPS13A-D*). Although mutations in all four *VPS13A-D* genes are linked to developmental or neurodegenerative diseases, such as chorea acanthocytosis (ChAc; *VPS13A*), Cohen syndrome (*VPS13B*), early onset Parkinson’s disease (*VPS13C*), and ataxia-paraplegia (*VPS13D*) [1,2,3,4], the functions of the proteins they encode have only recently begun to be uncovered. The first data relating to their molecular function came from a yeast study in which Vps13 was found to be present in several membrane contact sites (MCSs) [5,6,7]. MCSs are structures in which the membranes of two organelles are maintained in close proximity, but do not fuse [8]. These sites are responsible for lipid and ion transport between organelles, and are important for lipid biosynthesis and communication between organelles. Only recently, each of the four human VPS13 proteins was found to localize to a specific MCS. VPS13A was shown to be present at ER-mitochondria and at ER-lipid droplet MCSs [9], VPS13B at MCSs between recycling endosomes and early endosomes [10], and VPS13C at MCSs between the ER and endosomes [9] and VPS13D at ER-mitochondria, ER-peroxisome [11,12], and lipid droplet–mitochondria MCSs [13]. Additionally, VPS13B and VPS13D localize to the membranes of the Golgi apparatus (henceforth Golgi membranes), but the contact membrane was not determined [14,15]. Such localization patterns suggest that the function of the single yeast Vps13 in multiple MCSs has been divided across the multiple VPS13 paralogues.

VPS13 proteins have a characteristic structure with the most conserved regions at the N- and C-termini [16]. These regions contain a number of specific motifs and domains. The N-terminal part of VPS13 from *Chaetomium thermophilum* has recently been crystalized and it was shown that this region forms a channel/hydrophobic groove that could accommodate lipids [9,17]. The ability of the corresponding N-terminal region of yeast Vps13 to transfer lipids was demonstrated in vitro [9]. The central region and C-terminal domains, in the yeast Vps13: VAB, APT1, ATG_C, and Pleckstrin homology (PH)-like domains, are likely responsible for the localization of Vps13 to different MCSs through interaction with adaptor proteins and lipids [18]. For example, the interaction with Mcp1 is responsible for the recruitment of Vps13 to mitochondria [7] and interaction with phosphatidylinositol 3-phosphate (PI(3)P) likely participates in the recruitment of Vps13 to endosomes [19,20]. PH domains are generally thought to bind to inositol lipids and to proteins [21]. Indeed, a C-terminal fragment of Vps13, including the PH-like domain, was found to bind to phosphatidylinositol 4,5-bisphosphate (PI(4,5)P_2_) [22].

The removal of Vps13 from yeast cells by deletion of the *VPS13* gene causes pleiotropic changes in cell physiology. This includes defects in protein sorting at different stages of vesicular trafficking, resulting in secretion of vacuolar carboxypeptidase Y (CPY) [23], defects in endocytosis, and Sna3 protein trafficking [20]. Additionally, the *vps13*Δ mutant shows defects in sporulation [24], in the organization of the actin cytoskeleton [20], in calcium and iron homeostasis [25,26], and in mitochondrial function [6]. Some of these phenotypes are also observed in cells from patients who carry mutations in *VPS13* genes [14,27,28,29,30,31,32,33,34,35]. However, at present it is hard to find a link between the lipid transfer function of VPS13 proteins at specific locations, molecular phenotypes, and the manifestation of disease(s).

The Golgi is a polarized organelle. Newly synthesized cargos from the ER arrive at the cis-Golgi, are modified and sorted, and then leave via the trans-Golgi network (TGN). The major regulators of the flow of membranes and proteins, both inside and outside of the Golgi, are small GTPases of the ADP ribosylation factor (Arf) family. The Arf proteins perform their function through interaction with, and regulation of the activity of, several effector proteins that often possess PH domains responsible for Arf1 binding [36,37]. An increasing number of reports link changes in Golgi morphology to neurodegenerative disorders, including Alzheimer’s disease, Parkinson’s disease, and amyotrophic lateral sclerosis [38,39], but also to many other diseases [40,41]. There are also new studies showing the functional connections between the Golgi and mitochondria [42].

A study revealed that in several model organisms, the lack of Arf1 leads to mitochondrial hyperfusion [42,43]. However, the molecular mechanisms underlying these changes differ between organisms. In yeast cells, the lack of Arf1 causes an accumulation of the pro-fusion Fzo1 GTPase in mitochondria [43]. In contrast, in HeLa cells Arf1 does not affect the level of fusion/fission factors, but instead is required (along with phosphatidylinositol 4-phosphate, PI(4)P, in TGN vesicles) for the division of mitochondria at ER-induced mitochondria constriction sites [42,44].

The presence of a PH-like domain, which could be responsible for the interaction with Arf1 GTPase, and the fact that mutations in the yeast *VPS13* gene cause similar defects to *arf1* mutations, has prompted us to investigate whether there is a functional and/or physical link between the Arf1 GTPase and the Vps13 protein. We have found that Arf1 and Vps13 work in parallel in maintaining mitochondrial function and in endocytosis, but work together in Golgi membranes during the process of CPY sorting. Importantly, Vps13, through its PH-like domain, interacts with Arf1 and PI(4,5)P_2_, and this ability is also shared by the PH-like domain of VPS13A.

## 2. Results

### 2.1. Vps13 Co-Localizes with Clathrin Heavy Chain Protein and Influences the Number of Clathrin Spots

**Figure 1:**
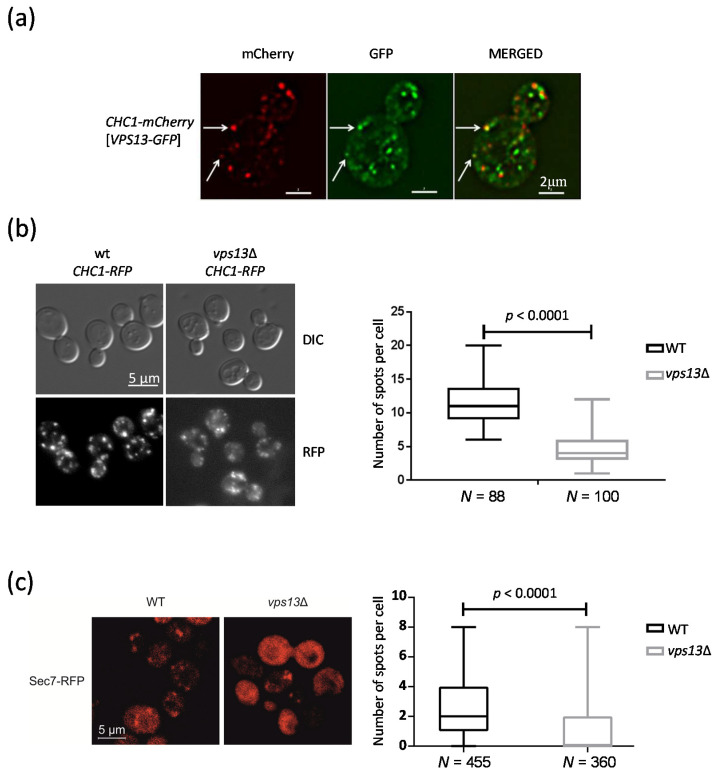
Vps13 co-localizes with Chc1 and the deletion of *VPS13* affects the number of clathrin and Sec7 spots. (**a**) Vps13 co-localizes with Chc1. Fluorescence microscopy images showing the localization of Chc1-mCherry and Vps13-GFP. The arrows indicate spots corresponding to both Vps13-GFP and Chc1-mCherry. (**b**) The *vps13*Δ mutant cells have a reduced number of Chc1-RFP spots compared to wild-type (WT) cells. WT cells or the *vps13*Δ cells expressing *CHC1-RFP* were observed by fluorescence microscopy and by differential interference contrast (DIC) microscopy. The chart illustrates the number of Chc1-RFP spots per cell in WT and *vps13*Δ mutant cells from three independent cultures. Test-*T*: *p* < 0.0001; (**c**) Sec7-RFP localization is altered in the *vps13*Δ mutant cells. WT and *vps13*Δ mutant strains were transformed with a plasmid encoding the Sec7-RFP fusion protein. The localization of Sec7-RFP was observed by fluorescence microscopy. The number of visible spots per cell was counted in three independent experiments. Test-*T*: *p* < 0.0001.

The first *vps13* mutant was isolated as secreting CPY [23]. Later, the direct requirement of Vps13 protein for clathrin-dependent transport from the Golgi to the endosomes was shown in vitro. The same study demonstrated a Vps13 requirement for homotypic fusion of the Golgi membranes in vitro [22]. We also showed that Vps13 is required for endocytosis [20]. Vps13 was also found in association with endosomes, recruited there by an interaction with the Ypt35 adaptor protein [18,45] or by an interaction with PI(3)P [19,20]. To further explore the role of Vps13 in trafficking, especially in the late secretory pathway and in endocytosis, we investigated the co-localization of Vps13 tagged with green fluorescence protein, Vps13-GFP, with clathrin. Clathrin is a complex built of light and heavy chains that forms a coat surrounding the emerging vesicle [46]. Clathrin is required at the Golgi for vesicular transport to endosomes (for cargos such as CPY) and in endocytosis [47,48], which are Vps13-dependent processes. As shown in Figure 1a, Vps13-GFP localized to internal punctate structures, some of which contained clathrin heavy chain (Chc1) protein tagged with mCherry, Chc1-mCherry. Next, we asked whether Vps13 is important for the formation of clathrin spots. To answer this question, we counted the number of clathrin spots (Chc1-RFP) that form in the wild-type (WT) and in *vps13*Δ mutant cells. In the *vps13*Δ cells, the number of spots was significantly reduced (Figure 1b), whereas the level of Chc1-RFP was not changed (Figure S1a). This finding is in agreement with a partial impairment of clathrin-dependent trafficking in the *vps13*Δ mutant [25,49,50].

### 2.2. Deletion of VPS13 Affects Sec7-RFP Localization

The formation of a clathrin coat is regulated by the small Arf1 GTPase, which initiates the recruitment of clathrin triskelions to membranes [51]. Importantly, Arf1 regulates transport from and within endosomes and the Golgi [52]. These processes are also dependent on the presence of functional Vps13. Thus, we suspected that Vps13 may be important for the function of Arf1 in these processes. To test this, we visualized, in WT and *vps13*Δ strains, the localization of Sec7-RFP, a guanine nucleotide exchange factor for Arf1, which is also a marker for the TGN. We observed a reduction in the number of Sec7-RFP spots in the *vps13*Δ strain in comparison to the WT strain (Figure 1c), whereas the level of Sec7 in both strains was similar (Figure S1b). This is in agreement with our earlier finding that less clathrin spots were formed in the *vps13*Δ strain. Vps13 likely influences the localization of Sec7 and thus the function of Arf1. This action may be (at least in part) responsible for the effects of the *vps13*Δ mutation on processes known to be dependent on Arf1.

### 2.3. The Vps13 Mutant Exhibits a Defect in Mitochondrial Function at an Elevated Temperature

To address the question of whether Arf1 and Vps13 participate in the same processes, and whether they do this together or in parallel, we analyzed the genetic interaction of *arf1* and *vps13* mutations by testing various phenotypes.

**Figure 2:**
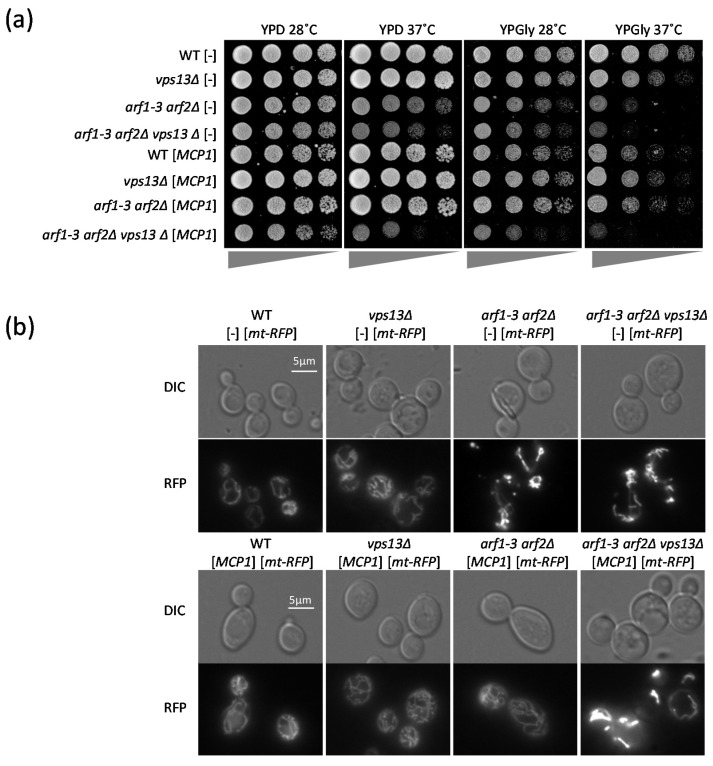
Vps13 and Arf1 influence mitochondria independently. (**a**) The *arf1-3 arf2*Δ *vps13*Δ strain shows a stronger growth defect in comparison to the *vps13*Δ or *arf1-3 arf2*Δ strains on media containing glycerol, a non-fermentable carbon source, and Vps13 is necessary for suppression of the growth defect of the *arf1-3 arf2*Δ strain by overexpression of the *MCP1* gene. The growth analysis of WT and the *vps13*Δ, *arf1-3 arf2*Δ, and *arf1-3 arf2*Δ *vps13*Δ strains, transformed with empty plasmid or carrying the *MCP1* gene, on YPD or YPGly media was carried out. Overnight liquid cultures of the indicated strains were diluted to the same density, serially diluted (4×), and spotted on glucose (YPD) or glycerol (YPGly) containing media. Plates were incubated at 28 °C or 37 °C for 2–5 days. (**b**) The Mcp1-dependent suppression of the mitochondrial morphology defect of the *arf1-3 arf2*Δ strain requires the presence of Vps13. The morphology of mitochondria was determined by fluorescence microscopy in WT and the *vps13*Δ, *arf1-3 arf2*Δ, and *arf1-3 arf2*Δ *vps13*Δ strains carrying plasmids encoding a red fluorescence protein targeted to the mitochondria (*mt-RFP*), a plasmid encoding Mcp1 (*MCP1*) or empty plasmid (-). Cells were also visualized by DIC microscopy.

Arf1 is important for mitochondrial function in cells grown on media containing fermentable or non-fermentable carbon sources [43]. Although a lack of *VPS13* affects mitochondria (it increases the frequency of mtDNA escape and the rate of mitophagy) and some *vps13* mutations suppress the *mmm1*∆ mutation (which results in the absence of the endoplasmic reticulum (ER)–mitochondria encounter structure (ERMES) complex, a complex responsible for the tethering of ER and mitochondria), neither changes in mitochondrial morphology nor growth defects on non-fermentable carbon source have been reported for *vps13*Δ cells. Thus, we tested the growth of *vps13*Δ on media containing glycerol (YPGly), a non-fermentable carbon source, at the regular temperature of 28 °C and when cells were grown at 37 °C, causing a higher demand for ATP. We found that *vps13*Δ grew more slowly on YPGly at 37 °C, suggesting a mitochondrial dysfunction (Figure 2a). This phenotype was used to test the genetic interaction between the *ARF* and *VPS13* genes. There are two *ARF* genes in yeast, *ARF1* and *ARF2* [53], and the deletion of each single gene is not deleterious for the cell. However, deletion of both is lethal. Thus, we used the *arf1-3 arf2*Δ strain, which carries a conditional allele, encoding Arf1-3, which is non-functional at an elevated temperature [54]. It grew more slowly on glucose-containing medium (YPD) at an elevated temperature (Figure 2a) and its growth on YPGly was reduced at 28 °C (Figure 2a), suggesting that Arf1-3 was already defective, which reduced mitochondrial function and limited growth. Next, we created the *arf1-3 arf2*Δ *vps13*Δ mutant strain. Its growth was tested on YPD and YPGly at 28 °C and at 37 °C. The growth of this triple mutant was slower compared to growth of the *arf1-3 arf2*Δ strain on YPD at 28 °C and on YPGly at 28 °C and 37 °C (Figure 2a). This indicates that the negative genetic interaction between *ARF1* and *VPS13* is additive and suggests that these two genes encode proteins from two parallel pathways that are important for mitochondrial function. A similar additive genetic interaction was observed for mtDNA escape in *arf1-3 arf2*Δ and *vps13*Δ, confirming that Arf1 and Vps13 influence mitochondrial processes in parallel (Figure S2a).

If Vps13 and Arf1 influence mitochondria independently, then Arf1 should not impinge on the recruitment of Vps13 to mitochondria by Mcp1, an integral protein of the outer mitochondrial membrane. We used the observation that Mcp1 overproduced from a plasmid causes Vps13 to be almost exclusively attached to mitochondria [7], and checked the localization of Vps13-GFP in the *vps13*Δ and *arf1-3 arf2*Δ *vps13*Δ strains transformed with plasmids encoding the Mcp1 protein. Analysis of the fluorescence microscopy images revealed that when *MCP1* was additionally expressed from the plasmid the Vps13-GFP protein was located around the mitochondria in *vps13*Δ cells, as expected. This was also the case for *arf1-3 arf2*Δ *vps13*Δ cells (Figure S2b). However, in these cells some Vps13-GFP-containing structures did not co-localize with mitochondrial staining, suggesting that the presence of Arf1 negatively influences the recruitment of Vps13 to other membrane(s). These results indicate that the presence of the Arf1 protein is not necessary for the Mcp1-dependent recruitment of Vps13 to the mitochondrial outer membrane.

In order to confirm that Vps13 really has an Arf1-independent effect on mitochondrial functions, we tested how overexpression of the *MCP1* gene influenced the growth of our strains of interest on YPGly. The overexpression of *MCP1* improved the growth of the *arf1-3 arf2*Δ strain on YPGly at both 28 °C and 37 °C (Figure 2a). Moreover, the presence of Vps13 was necessary for the *MCP1*-dependent suppression of this growth defect, because in the *arf1-3 arf2*Δ *vps13*Δ strain overexpression of *MCP1* did not improve growth (Figure 2a). Next, we asked whether overexpression of *MCP1* improves the morphology of mitochondria in the *arf1-3 arf2*Δ strain. To this end, the WT, *vps13*Δ, *arf1-3 arf2*Δ, and *arf1-3 arf2*Δ *vps13*Δ strains were transformed with a plasmid encoding mt-RFP, an RFP protein fused to a mitochondrial targeting sequence [55], and an empty plasmid, or a plasmid encoding Mcp1. Analysis of the images showed that overexpression of *MCP1* helped to restore normal mitochondrial morphology in the *arf1-3 arf2*Δ strain, but did not in the *arf1-3 arf2*Δ *vps13*Δ strain (Figure 2b). This means that Mcp1 rescues the morphology of mitochondria in an *arf1-3 arf2*Δ strain only in the presence of Vps13.

In summary, these results show that both Vps13 and Arf1 are important for mitochondrial functions, but that they participate in two separate, complementary pathways.

### 2.4. Vps13 and Arf1 Proteins Cooperate in the Sorting and Transport of Proteins from the Golgi

**Figure 3:**
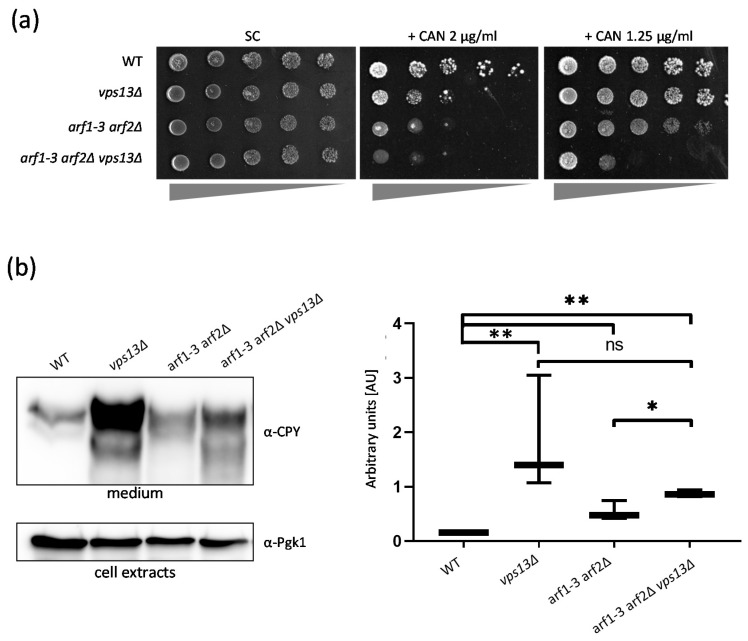
Vps13 and Arf1 proteins do not cooperate in endocytosis, but do cooperate in sorting proteins at the Golgi. (**a**) Sensitivity to L-canavanine (CAN) of WT and *vps13*Δ, *arf1-3 arf2*Δ, and *arf1-3 arf2*Δ *vps13*Δ strains. Serial (4×) dilutions of yeast cultures were spotted onto SC or SC-arg supplemented with CAN and incubated at 33 °C for 5 days. (**b**) Vps13 cooperates with Arf1 in CPY sorting at the Golgi. Overnight yeast cultures were diluted to OD_600_ 0.5, cultured in YPD medium at 35 °C for 1.5 h, and then harvested by centrifugation. The supernatants, containing secreted CPY, were concentrated on filters. Protein extracts from the cell pellets were obtained to determine the Pgk1 level (used as a control for the number of cells). Both fractions were analyzed by Western blot using α-CPY and α-Pgk1 antibodies. Experiments were performed in triplicate. Densitometry analysis of the blots was performed and presented in graphical form; (AU)—arbitrary units. Test-*T*: ns—non-significant; * *p* < 0.05; ** *p* < 0.01.

Next, we tested the genetic relationship between the *vps13*Δ and *arf1-3 arf2*Δ mutations in other processes. The endocytic defect of *vps13*Δ cells is the reason for hypersensitivity to canavanine, an arginine analogue [20], whereas the sorting defect causes an enhanced secretion of CPY [56]. The Arf1 GTPase, similar to Vps13, participates in endocytosis and protein vesicular transport [57]. Thus, it is likely that both of these proteins cooperate in these trafficking processes. A comparison of growth rates for WT, *vps13*Δ, *arf1-3 arf2*Δ, and *arf1-3 arf2*Δ *vps13*Δ strains on L-canavanine (CAN)-containing plates showed that *arf1-3 arf2*Δ is even more sensitive to this compound than the *vps13*Δ strain and that this sensitivity is increased further in the *arf1-3 arf2*Δ *vps13*Δ strain (Figure 3a). This finding suggests that Vps13 and the Arf1 GTPase work separately in endocytosis.

Next, secretion of CPY was evaluated. An equal number of cells of the WT, *vps13*Δ, *arf1-3 arf2*Δ, and *arf1-3 arf2*Δ *vps13*Δ strains were cultured in liquid media at 35 °C for 1.5 h. Spent media were collected and the level of secreted CPY was determined by Western blot. The level of secreted CPY in all mutant strains examined was higher than in the WT strain. However, the amount of CPY secreted by the *arf1-3 arf2*Δ *vps13*Δ strain was not higher than the amount observed for the *vps13*Δ strain (Figure 3b). This indicates that *ARF1* is epistatic to *VPS13* and that Arf1 and Vps13 work in the same pathway during CPY sorting at the Golgi. Based on the results presented here, we conclude that Vps13 and Arf1 most likely participate together in the Golgi in the process of protein sorting to endosomes, but work separately in endocytosis.

### 2.5. The PH-like Domains of Vps13 and VPS13A Proteins Interact Directly with the Arf1 GTPase

**Figure 4:**
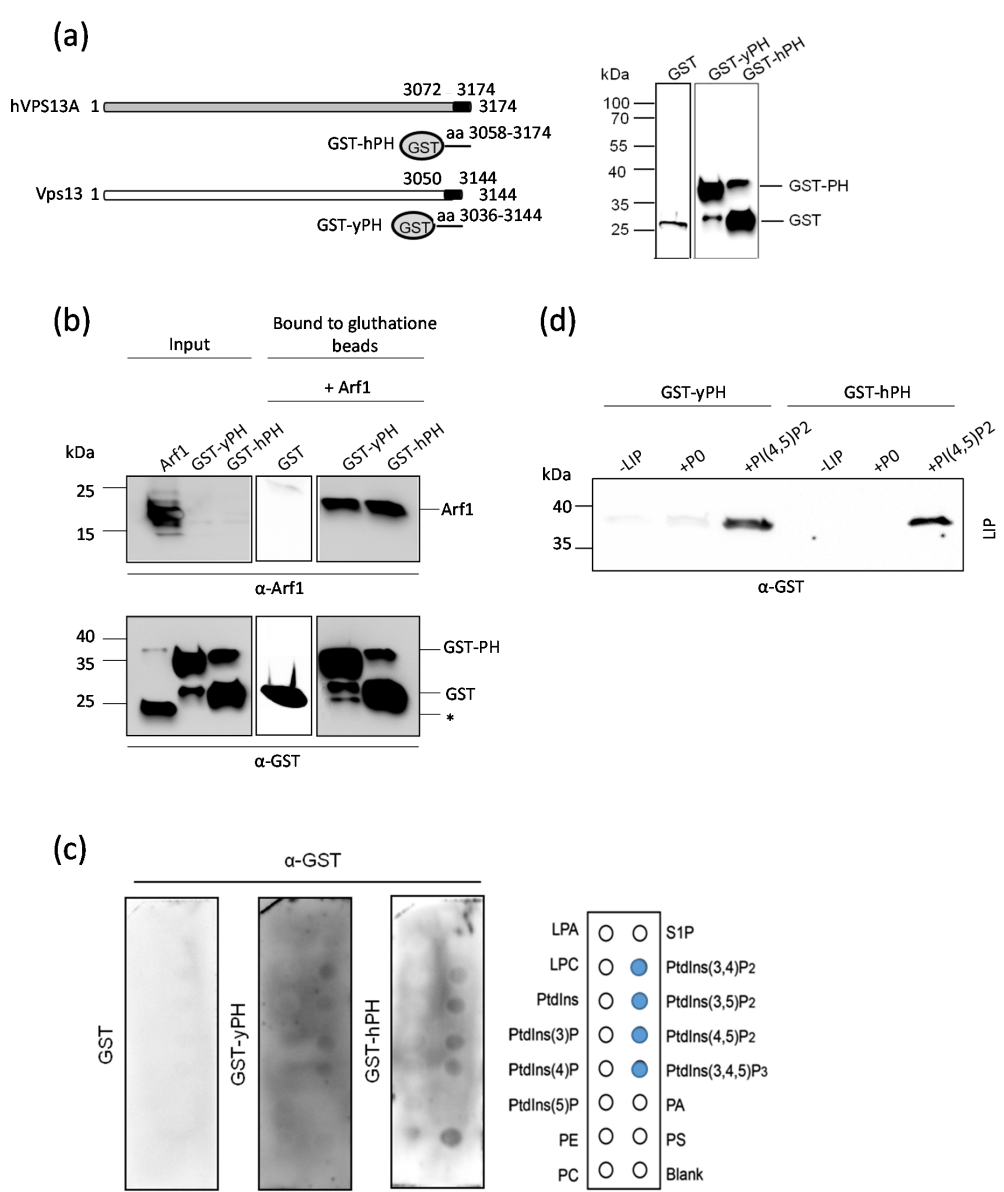
The interaction of PH-like domains from Vps13 and VPS13A with Arf1-GTP and selected phosphatidyl inositol phosphates in vitro. (**a**) Schematic representation of Vps13 and VPS13A and the fragments fused to GST that were used to test the binding to Arf1 GTPase and to lipids. The yPH and hPH domains are colored black. Western blot analyses of GST-yPH and GST-hPH purified from bacteria are shown in the right-hand panel. (**b**) Purified recombinant GST-yPH and GST-hPH or GST alone were bound to glutathione beads before the addition of recombinant Arf1. The purified proteins used in the experiment (input) and proteins bound to glutathione beads were analyzed by Western blot using α-Arf1 and α-GST antibodies; *—Non-specific band. (**c**) Analysis of lipid binding by yPH and hPH domains. Purified GST-yPH and GST-hPH proteins (1 µg each) were used to analyze the interaction with phospholipids on membranes by protein lipid overlay assay. Lipid-bound proteins were detected by Western blot using α-GST antibodies. GST protein was used as a negative control. (**d**) The yPH and hPH domains bound to liposomes enriched in PI(4,5)P_2_. Purified GST-yPH and GST-hPH proteins were incubated without liposomes (-LIP), with biotinylated liposomes without PIPs (P0) and with liposomes enriched with 5% PI(4,5)P_2_ (+PI(4,5)P_2_). Liposomes were collected using streptavidin-coated beads. Binding of proteins to liposomes was analyzed by Western blot using α-GST antibody (LIP).

The genetic interactions observed between the *VPS13* and *ARF* genes, in particular the likely involvement of Arf1/2 and Vps13 together in CPY sorting, led us to investigate whether these proteins interact physically. The PH-like domain of the yeast Vps13 protein (yPH) and that of VPS13A (hPH), as an example of PH domains of human VPS13, are potentially capable of interacting with a GTPase. To test this, fusion proteins consisting of the PH-like domains and glutathione S-transferase (GST), GST-yPH and GST-hPH (Figure 4a), were produced and purified from bacterial cells. Both of these proteins tend to degrade, with GST-hPH being less stable (Figure 4b). The interaction of these fusion proteins with Arf1 was tested by affinity chromatography. The purified fusion proteins were immobilized on glutathione-coated magnetic beads and then incubated with purified recombinant Arf1 and GTP (Arf1-GTP). The bound fraction was eluted and analyzed by Western blot using anti-GST and anti-Arf1 antibodies. The Arf1 protein (molecular mass ca. 20 kDa) was detected in the bound fractions with both fusion proteins, GST-yPH and GST-hPH. The slower migrating forms of Arf1 present in the purified fraction did not bind to immobilized GST-PH domains (Figure 4b). These results show the ability of the PH-like domains of Vps13 and VPS13A to interact directly with Arf1 and suggest that both of these proteins can be recruited to Arf1-containing membranes by their PH-like domains.

It is known from the literature that domains from the PH family also exhibit an ability to bind phospholipids [36,58]. We tested whether the PH-like domains present in Vps13 and VPS13A could interact with lipids, in addition to their interaction with Arf1. Purified recombinant GST-yPH and GST-hPH were tested for specific interactions with glycerophospholipids using a protein lipid overlay assay. Both yPH and hPH domains exhibited binding to phosphatidylinositol 3,4-bisphosphate (PI(3,4)P_2_), phosphatidylinositol 3,5-bisphosphate (PI(3,5)P_2_), PI(4,5)P_2_, and phosphatidylinositol 3,4,5-trisphosphate (PI(3,4,5)P_3_) (Figure 4c). These interactions appeared to be weak, similar to what has been described for many PH domains [58]. Therefore, we next investigated the binding of GST-yPH and GST-hPH to liposomes. Because earlier work by De et al. showed that the C-terminal part of Vps13 bound only to PI(4,5)P_2_ [22], we used liposomes enriched with this lipid_._ For controls, recombinant proteins were incubated with liposomes that were not enriched in PIPs (+P0) or without liposomes entirely (-LIP). Both GST-yPH and GST-hPH proteins bound efficiently to liposomes containing PI(4,5)P_2_, whereas no binding to control liposomes (P0) was observed (Figure 4c). This indicates that the PH-like domain is most likely responsible for the binding of the yeast Vps13 protein to PI(4,5)P_2_, and the C-terminal fragment of VPS13A also binds to this lipid.

In summary, the PH-like domains of Vps13 and VPS13A have a dual binding ability. They can bind to both Arf1 GTPase and to PI(4,5)P_2_.

### 2.6. The PH-like Domain of Vps13 Is Required for Interaction with Arf1 In Vivo and Drives Its “Passenger” GFP Protein to the Sec7-RFP-Positive Compartment

**Figure 5:**
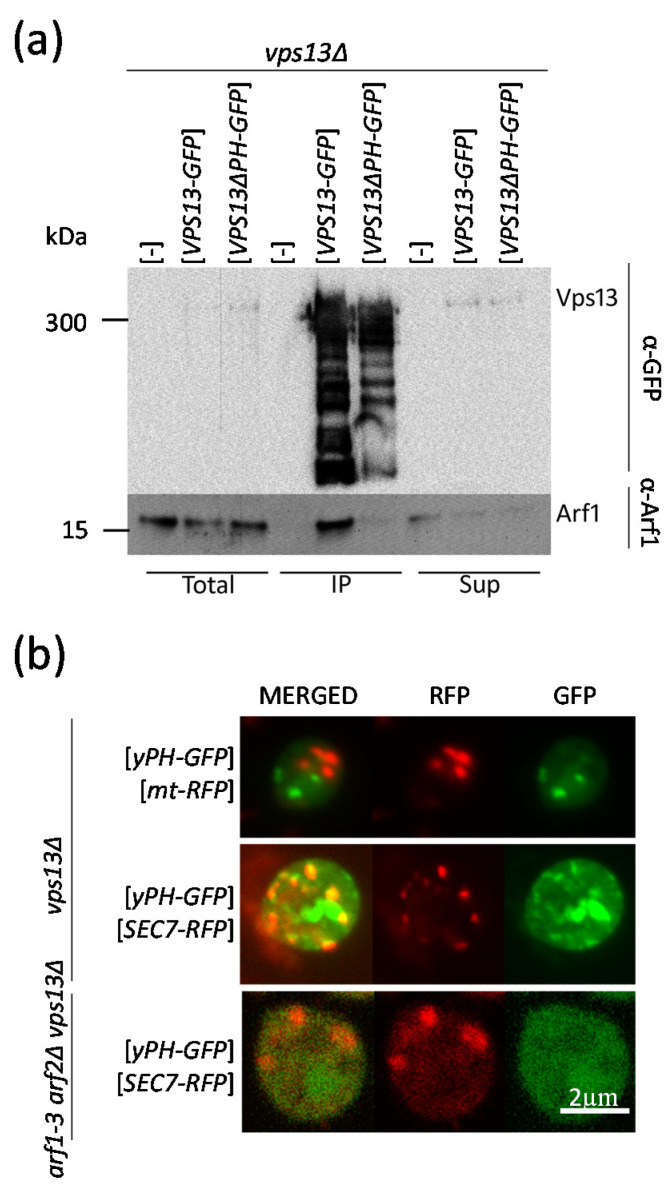
Vps13 interacts with Arf1 in vivo. (**a**) Cell extracts (total) from the *vps13*Δ strain transformed with an empty vector (-), a vector-expressing tagged Vps13 (*VPS13-GFP*) or a truncated, tagged version of Vps13 (*VPS13*∆*PH-GFP*) were obtained. The extracts (Total), the fraction of extracts bound to GFP trap beads (IP) and non-bound material (Sup) were analyzed by immunoblotting using anti-GFP and anti-Arf1 antibodies. (**b**) Analysis of yPH-GFP localization in relation to mitochondria and Golgi marked with mt-RFP or Sec7-RFP, respectively, in *vps13*Δ or *arf1-3 arf2*Δ *vps13*Δ strains observed by fluorescence microscopy.

To test whether the Vps13-Arf1 interaction also occurs in vivo, co-immunoprecipitation was performed. The *vps13*Δ strain was transformed with plasmids encoding full-length *VPS13*, *VPS13-GFP*, or *VPS13* devoid of a fragment encoding the PH-like domain, *VPS13*∆*PH-GFP*. Cell extracts were obtained and Vps13-GFP and Vps13ΔPH-GFP proteins were trapped on GFP-trap beads. The co-immunoprecipitated fractions containing Vps13 variants were analyzed by Western blot for the presence of Vps13 and Arf1. Both versions of GFP-tagged Vps13 were effectively trapped on beads (Figure 5a). The Arf1 protein was retained by Vps13-GFP and, substantially less efficiently, by Vps13ΔPH-GFP, but was not retained in a control strain without a tagged Vps13. Overall, our experiments confirmed that a direct interaction of Vps13 and Arf1 occurs also in vivo.

The physical interaction of the PH-like domain of Vps13 with a purified Arf1 GTPase in vitro and detection of the Arf1-Vps13 interaction in vivo led us to investigate whether the PH-like domain could be localized to Arf1-containing organelles in vivo. A plasmid containing a sequence coding for a fragment of Vps13, corresponding to the PH-like domain fused with GFP (yPH-GFP), was created and transformed into *vps13*Δ cells. The *vps13*Δ strain was used in order to avoid competition between yPH-GFP and the endogenously encoded full-length Vps13. As determined by fluorescence microscopy, the fusion protein yPH-GFP localized to the punctate structures. We tested whether these structures corresponded to mitochondria or Golgi, the expected localizations of Arf1 GTPase. These organelles were visualized using the mt-RFP fusion and Sec7-RFP, respectively. We decided to detect Sec7-RFP even though in most of the *vps13*Δ cells the Sec7 is dispersed in the cytoplasm (Figure 1c). There was no co-localization of yPH-GFP with mt-RFP (Figure 5b). Significantly, the fluorescence from yPH-GFP overlapped with the signal from Sec7-RFP in cells in which Sec7 localized to spots (Figure 5b). We also asked whether Arf1/2 proteins are necessary for yPH localization to the Sec7-mRFP-containing structures. To answer this question, the localization of yPH-GFP was also analyzed in the *arf1-3 arf2*Δ *vps13*Δ strain. In this strain, yPH-GFP was dispersed in the cytoplasm, even in cells in which Sec7-mRFP was in spots (Figure 5b), meaning that yPH-GFP is not localized to Sec7-containing structures due to a lack of functional Arf1/2 proteins.

From these results, we conclude that the Vps13 interaction with Arf1 is direct, requires the PH-like domain of Vps13, and occurs in vivo at Golgi membranes.

## 3. Discussion

VPS13 proteins are most probably responsible for lipid transfer at MCSs. A lack of VPS13A-D proteins results in specific developmental and neurodegenerative diseases. Thus, it is important to understand the mechanisms by which each of these proteins is recruited to specific types of MCSs. The single yeast Vps13 protein is present at multiple different MCSs; by studying its localization we can orient our search for the determinants and regulatory signals responsible for the localization of VPS13 to the membranes of specific organelles. Here, by genetic analysis we showed that the Arf1 GTPase and Vps13 participate together in sorting of CPY, whereas in other Arf1- and Vps13-dependent processes, these proteins work in parallel. Furthermore, we characterized the PH-like domain of Vps13 as being responsible for the localization of Vps13 to Golgi membranes through an interaction with Arf1. We also showed that the PH-like domain interacts with a lipid, PI(4,5)P_2_. Finally, we demonstrated that the ability to bind Arf1 GTPase and lipids is also exhibited by the PH-like domain of VPS13A. Thus, in addition to lipids and protein adaptors, we identified Arf1 GTPase as a factor that determines the localization of VPS13 proteins to specific organelles. These findings will help to better understand the pathogenesis of VPS13-related diseases.

The importance of VPS13 proteins for mitochondrial function is well documented [33]. Here, we have demonstrated that a weak growth defect of the *vps13*∆ strain on a non-fermentable carbon source can be observed, but only at an elevated temperature. We have also shown that the importance of Vps13 for mitochondrial functions is more easily detectable when the Arf1/2 proteins are defective. In most cases, an additive genetic interaction occurs when interacting genes encode proteins from two parallel pathways participating in the same process [59]. Previously, Arf1 was shown to be involved in mitochondrial function in yeast, and was found in the mitochondrial fraction of yeast cell extracts [43]. Thus, the negative genetic interaction that we observed between *ARF1/2* and *VPS13* mutations explains why no changes in mitochondria shape or growth on non-fermentable carbon sources have been described for the *vps13*∆ mutant until now, because the Arf1/2-dependent pathway is able to complement the lack of Vps13 on mitochondria. However, deletion of the *VPS13* gene results in the escape of mtDNA to the nucleus and in increased mitophagy [6], and Vps13-GFP was observed on mitochondrial membranes, recruited by interaction with Mcp1. The necessity of Vps13 for the *MCP1*-based suppression of *arf1-ts arf2*∆ mitochondrial phenotypes supports one interpretation, namely, that Vps13 and Arf1 act in two parallel pathways related to mitochondrial functions (despite the fact that these two proteins can interact directly). However, how do Arf1 and Vps13 influence mitochondria? Firstly, in yeast cells, Arf1 is required for efficient PI4P production by Pik1 kinase and in a *pik1* mutant, the PI4P level is reduced to 50%. Also, a lack of Vps13 reduces the level of PI4P, but by an unknown mechanism. Thus, the effect of the *arf1-ts arf2*∆ and *vps13*∆ mutations on the reduction of PI4P levels could be additive. It was demonstrated in HeLa cells that Arf1 regulates PI4P production and that both Arf1 and PI4P are necessary for proper mitochondria fission [42]. However, there is no evidence about the influence of PI4P on mitochondria in yeast. The second possibility is that Arf1 and Vps13 proteins participate in the formation of alternative MCSs that are important for mitochondrial functions. In *Candida*, the *arf1*∆/*arf1*∆ mutant was shown to have abnormal mitochondrial morphology and extensive ERMES formation [60]. We found that the changes in mitochondrial morphology observed in *arf1-ts arf2*∆ could be suppressed by overexpression of *MCP1*, which required the presence of Vps13. This is intriguing because on the one hand, *MCP1* overexpression could suppress the lack of ERMES [61]. On the other hand, *MCP1* overexpression could also suppress the mitochondrial phenotypes of the *arf1-ts arf2*∆ strain, which is expected to have extensive ERMES formation [60]. Moreover, the effect of *MCP1* overexpression in the *mmm1*∆ mutant is linked to the formation of connections between the vacuole and mitochondria and involves Vps13 [7]. Thus, we conclude that Vps13 acts in a pathway parallel to Arf1 by the formation of MCSs.

In yeast, Vps13 is necessary for the proper sorting of CPY in the Golgi [62] and is directly required for Golgi vesicle formation and for homotypic fusion of Golgi membranes [22]. Here we showed that Vps13 co-localizes to structures containing clathrin, a coat complex that can form on TGN membranes [63,64]. In humans, two paralogs—VPS13B and D—have been found on Golgi membranes [11,14], but only in the case of VPS13B is it known that the localization to the Golgi is mediated by an interaction with the GTPase Rab6 [14]. In the case of yeast Vps13, such a localization can be achieved through the interaction of the Vps13 VAB domain with currently unknown adapter protein(s), similarly to the recruitment of Vps13 to endosomes by Ypt35, to prospore membrane by Spo71, and to mitochondria by Mcp1 [7,24]. The interaction between Vps13 and the Golgi membranes could also be established through Golgi-specific lipids. Purified Vps13 binds well to liposomes containing PI(4)P, the lipid produced and enriched in Golgi membranes [65,66]; the N-terminal region of Vps13 is responsible for this interaction [22]. We recently demonstrated that Vps13 and VPS13A possess the APT1 domain [19,20]. This domain was first identified in the *Zea mays* APT1 protein, which co-localized with a Golgi marker in vivo [67]. Very recently, the Apt1 domain of the *Drosophila* Hobbit protein, the homolog of the *Z. mays* APT1 protein, was found to bind several lipids: PI, PI(4)P, PI(4,5)P_2_, and PI(3,4,5)P_3_ [68], similarly to the APT1 domains of Vps13 and VPS13A, which, in addition to binding to PI(3)P and PI(5)P, could bind PI(4)P [19]. Thus, the Golgi localization of Vps13 could be achieved through the interaction of APT1 with PI(4)P. However, the interaction between Vps13 and Arf1, mediated by the C-terminal PH-like domain of Vps13, could also contribute. This interaction may also be necessary for proper vesicular trafficking at the Golgi and between membranes of the endo-lysosomal system. In support of this, cells producing a Vps13 protein lacking the PH-like domain, similarly to the *vps13*Δ, exhibit an increased level of CPY secretion compared to wild-type cells [69]. It should also be noted that the absence of Vps13 results in other phenotypes similar to those caused by a lack of Arf1, such as a reduced number of structures containing Sec7-mRFP or clathrin. It was demonstrated that Arf1 is important for Sec7 binding to membranes [70] and indirectly participates in the recruitment of clathrin by binding-coat adaptors [71]. Together, the phenotypes of the *vps13*Δ strain might simply occur because of the lack of Arf1-Vps13 complex formation and a reduction of Arf1 activity on Golgi membranes. Why, then, is CPY secretion less efficient in *arf1-3 arf2*∆ *vps13*Δ than in the *vps13*Δ strain? This could be explained as a consequence of lower GTPase activity in the *arf1-3 arf2*∆ strain compared to the *vps13*Δ strain. In the cascade of sorting events that take place at the Golgi, CPY exit precedes the formation of secretory vesicles [72,73]. Thus, *arf1-3 arf2*∆ mutations either reduce the availability of CPY for sorting into vacuole directed vesicles or block CPY secretion at a later stage. Altogether, our results support the view that the PH-like domain is responsible for binding Vps13 to Golgi membranes through an interaction with Arf1 and that this is important for CPY sorting.

The interaction of the C-terminal part of Vps13 with PI(4,5)P_2_-containing liposomes has previously been shown [22]. Here, we narrowed the region responsible for this interaction to the PH-like domain. However, whereas Arf1 is associated with the Golgi and with endosomes, PI(4,5)P_2_ is a lipid characteristic of the plasma membrane. PI(4,5)P_2_ is produced at the plasma membrane from PI(4)P by a PI(4)P 5-kinase, the Mss4 protein [74]. The importance of the interaction between Vps13 and PI(4,5)P_2_ is unknown, but it could be involved in endocytosis and the organization of the actin cytoskeleton [20]. The other possibility is that PI(4,5)P_2_ binding occurs on other membranes, as this lipid, although in lower amounts, is present in other membranes where it is important for processes such as exocytosis and autophagy [75]. Importantly, the dual-binding specificity of PH domains for lipids and Arf1 was already described. The paradigm is the PH domain of the yeast Osh1 protein, which is involved in lipid transport [37,76]. Osh1 binds to both PI4P and Arf1. Moreover, the lipid-binding specificity of PH domains could be independent of their association with the Golgi [76], because the PH domains of different proteins are able to bind to the same lipid localized to different membranes. Thus, it is possible that the PH-like domain of VPS13 proteins can bind, independently of Arf1, to the Golgi and to PI(4,5)P_2_ in other localizations.

Interestingly, VPS13 proteins interact with various GTPases: VPS13B with Rab6 [14], VPS13C with Rab7 [77], and VPS13D with Miro [11]. Here we showed a direct interaction between the PH-like domain of VPS13A and the Arf1 GTPase. This interaction is especially intriguing because the Golgi localization of VPS13A has not previously been reported. The interactions between VPS13 proteins and GTPases, the master regulators of membrane processes that rely on membrane remodeling, suggest that VPS13 proteins could be effectors, recruited to provide the necessary lipids. Further studies will be necessary to answer important questions that remain: (1) Are VPS13 proteins really Arf effectors, or are Arfs regulated by VPS13? Alternatively, is it a mutual relationship? (2) Are the relationships between various VPS13 proteins and GTPases the same?

In conclusion, finding the interactions between Arf1 GTPase, Vps13, and the PH-like domain of VPS13A is important for a better understanding of the pathogenesis of diseases caused by mutations in *VPS13* genes. The Golgi is a central hub for protein sorting, which is important for neurodevelopment and for secretion of neurotransmitters, and its dysfunction is linked to several neurodegenerative disorders [38,40,41]. *ARF1* gene mutations are linked to autosomal dominant periventricular nodular heterotopia, a disorder characterized by delayed psychomotor development [78]. Moreover, changes in the regulation of Arf1, such as, for example, those caused by mutations in *ARFGAP2* encoding the Arf1 activating protein, manifest in chorea or microcephaly [79], the symptoms present in ChAc patients and Cohen syndrome patients. Therefore, it would be interesting to study the physical and functional connection between the VPS13 proteins and Arf1 GTPase in human cells in the future.

## 4. Materials and Methods

### 4.1. Strains, Plasmids, Media, and Growth Conditions

For cloning and plasmid propagation, *E. coli* DH5α was used. The yeast strains used in this study are listed in Table S1.

The strains *vps13*Δ::*natMX* and DKARF1 were constructed by integration of the *vps13::natMX* cassette into BY4741 and RT364 [54], respectively, and the strain *arf11*Δ::*natMX* was constructed by integration of the *arf1::natMX* cassette into BY4741 according to Goldstein and McCusker [80]. Transformants were selected on YPD + nourseothricin (NAT, WERNER BioAgents GmbH, Jena, Germany) plates. Integrations were confirmed by PCR of genomic DNA.

To construct strains for the mtDNA escape assay, a mtDNA donor *kar1-1* strain was constructed to facilitate the transfer of mtDNA from the PTY44 strain [81] to MATa *rho*^0^ strains. To do so, the strain JC8/55 [82] was crossed to PTY44 [81] as described [83]. Mating mixtures were diluted 1000× and plated on SC medium supplemented with leucine (at 20 mg L^−1^) and canavanine (at 20 mg L^−1^). Two independent respiring cytoductants were selected. In the second step, cytoductants were crossed to DFS160 [84] and 2 independent respiring clones, called AJT3 and AJT4, were selected. In parallel, the *trp1*Δ::*loxP-kanMX-loxP* cassette was integrated into following strains: BY4741, RT364, DKARF1, *vps13*Δ::*natMX*, and *arf1*Δ::*natMX*. In a subsequent step, all obtained Trp^−^ strains were treated with ethidium bromide to remove their mtDNA. For this purpose, the strains were passaged twice in YPD medium containing ethidium bromide at 40 μg mL^−1^. Cells from the last passages were spread for single colonies on YPD plates and incubated for 3 days at 28 °C. One non-respiring colony was picked for each Trp^−^ ethidium bromide-treated culture. Finally, mtDNA from AJT3 or AJT4 donor strains were transferred to Trp^−^ *rho*^0^ strains by crossing followed by a selection of respiring clones with the nuclear genomes of Trp^−^ strains to obtain AJT7, AJT9, AJT11, and AJT13.

The plasmids used in this study are listed in Table S2.The plasmid encoding *MCP1* was created by amplification of *MCP1* ORF using genomic DNA from BY4741 as a template and cloned into pRS416-P_GPD_ [85]_._ To create plasmids GST-yPH and GST-hPH the corresponding fragments of the *VPS13* and *VPS13A* genes were amplified by PCR using plasmids p415-P_TEF1_-VPS13 and p415-P_TEF1_-VPS13A, respectively, as templates and cloned into pKF463 [86]. The plasmid bearing VPS13-∆PH-GFP was constructed by one-step site-directed mutagenesis. pBluescript2SK+ plasmid with a 2388-bp SalI/SalI fragment from pUG35-VPS13 was amplified by PCR. The PCR product measuring approximately 5000 bps and lacking a 326-bps fragment coding for yPH was self-ligated. After isolation, the plasmid was digested with SalI endonuclease and a fragment of 2062 bps was cloned into p415-P_TEF1_-VPS13-GFP SalI/SalI.

The media used were YPD complete medium (1% yeast extract, 2% peptone, 2% glucose), YPD + NAT supplemented with 100 µg mL^−1^ of NAT, YPGly (1% yeast extract, 2% peptone, 3% glycerol), or synthetic complete (SC) medium (0.67% yeast nitrogen base without amino acids, 2% glucose) or SCGlyEtOH (0.67% yeast nitrogen base without amino acids, 3% glycerol, 3% ethanol) with required supplements as indicated.

For growth tests, transformants were grown overnight in liquid media, and cultures were diluted to an optical density OD_600_ of ~1. Four-fold serial dilutions of cells were spotted on solid YPD, YPGly, or SC–arg media, with canavanine added at the concentrations of 2 or 1.25 µg mL^−1^ (Sigma-Aldrich, St. Louis, MO, USA) as described in the figure legends. Plates were incubated at 28, 30, or 37 °C. For the mtDNA escape assay, 2 independent isolates of WT strain, *arf1-3 arf2- arf1-3 arf2- vps13*Δ::*natMX*, each lacking the nuclear copy *TRP1* gene but harboring mtDNA (*rho+, TRP1*), were grown on SCGlyEtOH plates at 30 °C for 2 days and then replica plated on SC–trp and incubated for 7 days at 30 °C. The number of papillae growing in each sector reflects the frequency of transfer of mtDNA to the nucleus for a given strain [81].

### 4.2. Microscopy

The co-localization of Vps13-GFP and Chc1-mCherry was performed in strain *CHC1-mCherry* [87] transformed with p415-P_TEF1_-VPS13-GFP. Cells were grown in SC–leu to logarithmic phase at 28 °C. Cells were observed using an Olympus IX-81 inverted fluorescence microscope (Olympus, Tokyo, Japan) with DeltaVision RT Restoration Microscopy (GE Healthcare, Little Chalfont, UK) and a Coolsnap HQ camera (Photometrics, Tucson, AZ, USA). Images were collected using SoftWoRx (GE Healthcare).

To observe the Sec7-RFP spots, BY4741 and *vps13*Δ strains carrying a plasmid containing the *SEC7-mRFP* gene [88] were grown overnight in SC–ura media at 28 °C. Cultures were diluted (1:10) in fresh SC–ura medium and allowed to grow for 4 h. Cells were collected by centrifugation, fixed by incubation for 25 min in 4% formaldehyde, and washed with KiPO4/sorbitol buffer (100 mM potassium phosphate pH 7.5 and 1.2 M sorbitol). Fixed cultures were spotted onto poly-L-lysine- or concanavalin A-coated slides, washed with water, and mounted in a mounting medium (Dako, Glostrup, Denmark). Cells were viewed using an LSM 780 Axio Observer Z.1 confocal microscope (Zeiss, Oberkochen, Germany). Images were collected using Zen 2012 black edition software (Zeiss). The confocal microscopy observations were performed in the Laboratory of Advanced Microscopy Techniques, Mossakowski Medical Research Institute, PAS. At least 100 cells were counted for every experimental variant.

To observe the morphology of mitochondria stained with MitoTracker Red, yeast cells were grown in SC–ura–leu until log phase growth. Then cells were transferred to SCGly–leu–ura medium and grown for 4 h at 35 °C, then centrifuged and suspended in SCGly–leu–ura medium supplemented with 25 nM MitoTracker Red CMXRos (Thermo Fisher Scientific, Waltham, MA, USA), incubated 15 min at room temperature, washed, and observed.

The Chc1-RFP, mt-RFP, and Vps13-GFP localization was observed and visualization of mitochondria stained with MitoTracker was performed in live cells using an Eclipse E800 fluorescence microscope (Nikon, Tokyo, Japan) equipped with a DS-5Mc camera (Nikon). Images were collected using the Lucia General 5.1 software (Laboratory Imaging Ltd., Praha, Czech Republic). The same fields were viewed by differential interference contrast (DIC) microscopy.

Images were processed using Adobe Photoshop 8.0 or GIMP 2.10. Graphs and statistical analyses were made in GraphPad Prism ver.8.0 (https://graphpad.com accessed on 20 April 2020).

### 4.3. Lipid Binding Assays

To identify lipids bound by the yPH and hPH domains, GST-fused proteins were produced and purified from bacteria. Membranes containing the arranged set of spotted phospholipids (PIPstrips; Echelon Biosciences Inc., Salt Lake City, UT, USA) were blocked with 3% fatty acid free BSA (Sigma-Aldrich) in TBST (TBS + 0.1% Tween-20) for 1 h at room temperature. Then 0.5–1 µg mL^−1^ of the purified GST-yPH and GST-hPH proteins or GST alone (a negative control) were incubated with prepared membranes in blocking buffer overnight at 4 °C. Membranes were washed in TBST and incubated with HRP-conjugated α-GST antibodies (BioLegend, San Diego, CA, USA). The chemiluminescence signal was captured using a CCD camera.

Quantitative analysis of protein–lipid interactions was performed using biotin-labeled synthetic liposomes containing 5% PI(4,5)P_2_, 65% phosphatidylcholine (PC), 29% phosphatidylethanolamine (PE), and 1% biotin (Echelon Biosciences Inc., Salt Lake City, UT, USA). Purified GST-yPH, GST-hPH, or GST alone as a negative control were incubated with 5 µL of liposome suspension enriched with PI(4.5)P_2_ and control liposomes no PIP-enriched (+P0) or no liposomes (-LIP) in 0.8 mL reaction buffer (50 mM HEPES, pH 7.5; 150 mM NaCl) with 2 mM EDTA, 1 mM CaCl_2_ for 30 min at room temperature. Then 50 μL of Dynabeads MyOne Streptavidin C1 (Invitrogen, Carlsbad, CA, USA) was added and incubated for 1 h at 4 °C. After a series of washes with the reaction buffer, the 2× sample buffer (120 mM Tris-HCl pH 6.8; 2% SDS; 20% glycerol; 0.04% bromophenol blue; 10% β-mercaptoethanol; 8 M urea) was added and samples were resolved on SDS-PAGE and analyzed by Western blot using HRP-conjugated α-GST antibodies (BioLegend). The bands on the membranes were subjected to densitometric analysis using the ImageJ program (https://imagej.nih.gov/ij/ accessed on 15 May 2020). Graphs and statistical analyses were made in GraphPad Prism ver.8.0 (https://graphpad.com accessed on 20 April 2020).

### 4.4. Analysis of yPH and hPH Interaction with Arf1 In Vitro

The yPH and hPH proteins were produced as N-terminally GST-labelled recombinant proteins (GST-yPH, GST-hPH) in the *E. coli* SoluBL21 (DE3) strain grown at 28 °C in LB medium supplemented with 0.2% glucose and 100 µg mL^−1^ ampicillin (Sigma-Aldrich). Protein expression was induced with 0.1 mM IPTG for 1 h at 28 °C. After induction, cells were centrifuged, resuspended in equilibration buffer (125 mM Tris-HCl pH 7.5, 150 mM NaCl) supplemented with a cocktail of protease inhibitors (cOmplete Mini, EDTA-free, Roche, Basel, Switzerland), and lysed by sonication. The homogenate was centrifuged at 20,000× *g* for 15 min at 4 °C. The supernatant was supplemented with 1 mM DL-dithiothreitol (DTT) and incubated in the presence of glutathione magnetic beads (Thermo Fisher Scientific) for 2 h at 4 °C. A total of 5 µg of purified Arf1 protein (courtesy of Anne Spang, Biozentrum, Basel, Switzerland) supplemented with 100 nmol GTP (Thermo Fisher Scientific) in washing buffer (125 mM Tris-HCl, 150 mM NaCl, 1 mM DTT, pH 7.5) was then added and the mixture was incubated for 1 h at room temperature, then rinsed several times. Bound proteins were eluted with the elution buffer (25 mM reduced glutathione, 125 mM Tris HCl pH 8.0, 150 mM NaCl, 1 mM DTT). Elution fractions and remaining fractions were used for Western blot analysis using the α-Arf1 (Anne Spang, Biozentrum University of Basel, Switzerland), α-GST-HRP (Biolegend, San Diego, CA, USA), and secondary α-rabbit HRP-conjugated (Dako) antibodies.

### 4.5. Co-Immunoprecipitation of Vps13 and Arf1

The transformants of the *vps13*Δ strain were grown in 300 mL of SC-leu to OD_600_ ~0.3–0.4 and 100 OD_600_ units were collected and resuspended in 250 µL of TNET buffer (10 mM TRIS pH 7.5, 1 mM EDTA, 150 mM NaCl, 0.1% Triton-X100) supplemented with an EDTA-free protease inhibitor cocktail (cOmplete Tablets; Roche). Cell extracts were prepared by disruption with glass beads via 10 min vortexing at 4 °C. The extracts were centrifuged at 300× *g* for 5 min at 4 °C. A total of 10 µL of obtained extract was precipitated with trichloroacetic acid (TCA) as a total fraction, and 100 µL of protein extract was then incubated with 25 µL of GFP Trap^®^ Agarose beads (ChromoTek, Planegg-Martinsried, Germany) for 1 h at 4 °C. After that time, 10 µL of the non-bound fraction was TCA precipitated as supernatant. The beads were washed 3 times with TNET buffer and transferred to new tubes following the second wash. The proteins bound to beads were eluted with 25 µL of 2× sample buffer (120 mM Tris-HCl pH 6.8; 2% SDS; 20% glycerol; 0.04% bromophenol blue; 10% β-mercaptoethanol; 8 M urea), boiled for 5 min, and resolved on TruPAGE™ Precast Gels, 4–20% (Sigma-Aldrich). Western blot analysis was performed using the α-Arf1 and α-GFP (Merck Life Science, Darmstadt, Germany) and secondary α-rabbit or α-mouse HRP-conjugated antibodies.

### 4.6. CPY Secretion

For quantitative analysis of the separated CPY, yeast strains were grown in YPD medium overnight at 28 °C to OD_600_ ~0.5 and then shifted for 1.5 h to 35 °C. The culture was centrifuged and the supernatant concentrated on Amicon filters (Merck). To the concentrated supernatants and pellets, 2× sample buffer (120 mM Tris-HCl pH 6.8; 2% SDS; 20% glycerol; 0.04% bromophenol blue; 10% β-mercaptoethanol; 8 M urea) was added. Samples were analyzed by Western blot preceded by SDS-PAGE. The antibodies used were α-CPY (Thermo Fisher Scientific), α-Pgk1 (Thermo Fisher Scientific), and a secondary α-mouse HRP-conjugated antibody. Densitometric analysis of Western blot bands was performed using ImageJ (https://imagej.nih.gov/ij/ accessed on 15 May 2020). Graphs and statistical analysis were carried out in GraphPad Prism ver.8.0 (https://graphpad.com accessed on 20 April 2020).

## Data Availability

The data that support the findings of this study are available from the corresponding authors upon request.

## Supplementary Materials

Supplementary Materials can be found at www.mdpi.com/article/10.3390/ijms222212274/s1.
