# Supplementary material for: The GTPase Arf1 Is a Determinant of Yeast Vps13 Localization to the Golgi Apparatus
Source: Int J Mol Sci. 2021 Nov 12;22(22):12274. doi: 10.3390/ijms222212274 (PMC8619211; doi:10.3390/ijms222212274)
Supplement: Supplementary file 1 [file ijms-22-12274-s001.zip › ijms-1440214-supplementary.pdf]

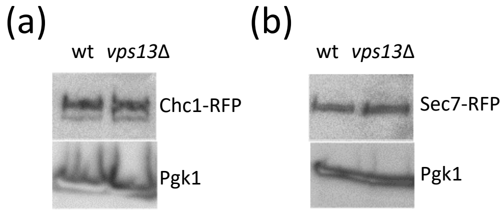

Figure S1. The levels of Chc1-RFP and Sec7-RFP are not changed in *vps13*Δ mutant. (**a**) The level of Chc1-RFP from protein extracts from the wild type and *vps13*Δ mutant cells was analysed by Western blotting with anti RFP antibodies. (**b**) The level of Sec7-RFP in protein extracts from the wild type and *vps13*Δ mutant cells transformed with plasmid encoding Sec7-RFP was analysed by Western blotting with anti RFP antibodies. The level of Pgk1 was determined as a control.

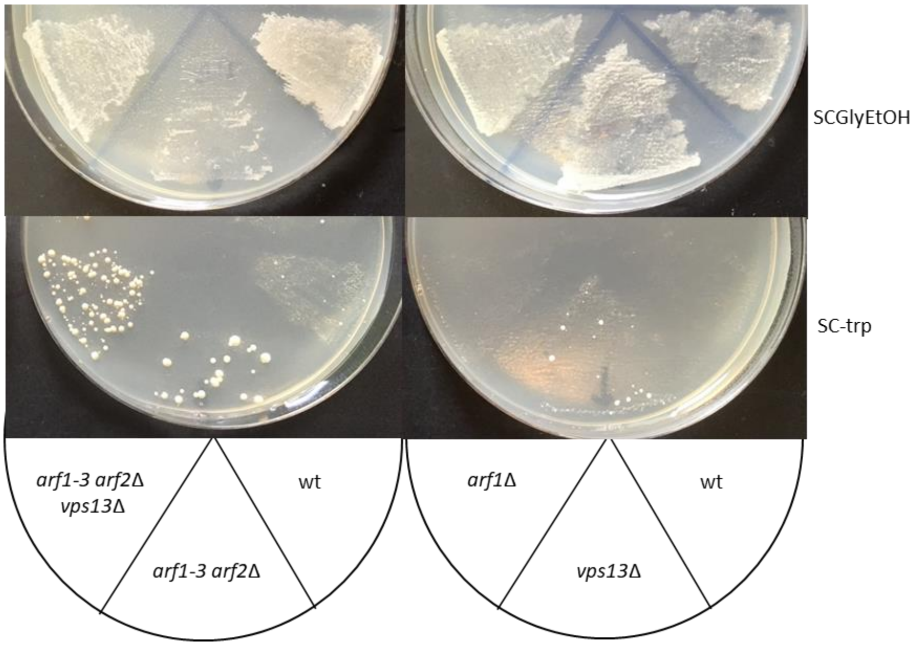

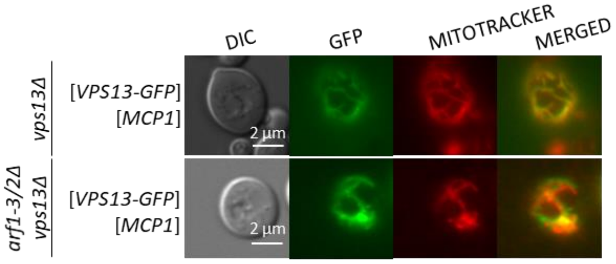

Figure S2. (**a**) *arf1-3 arf2Δ* and *vps13Δ* show additive effects on the rate of mtDNA escape. The strains AJT5, AJT7, AJT9, AJT11 and AJT13 were grown on SCGlyEtOH at 30 °C for 2 days and then replica-plated on to SC–trp and incubated for 5 days at 30 °C. (**b**) The Arf1 and Arf2 proteins are not necessary for Vps13 recruitment to mitochondrial membranes when *MCP1* is overexpressed. Fluorescence microscopy images of the *vps13Δ* and *arf1-3 arf2Δ vps13Δ* cells transformed with plasmids encoding Vps13-GFP and Mcp1. Transformants were grown in synthetic complete media (SC)–leu–ura and shifted to SCGly–leu–ura and incubated at 35 °C for 4 h. Mitochondria were visualized with MitoTracker and morphology of the cells was visualized by DIC microscopy.

Table S1. The strains used in this study.

Strain	Genotype	Reference
BY4741	MAT**a** *his3Δ1 leu2Δ0 met15Δ0 ura3Δ0*	Open Biosystems, Lafayette, CO, USA
vps13Δ	BY4741 *vps13Δ::KanMX*	Open Biosystems
vps13Δ:: natMX	BY4741 *vps13Δ::natMX*	This work
arf1Δ	BY4741 *arf1Δ::natMX*	This work
WR14	BY4741 *MET15*	This work
WR15	BY4741 *vps13Δ::KanMX MET15*	This work
RT364	MAT*α his3-Δ200 leu2-3,112 lys2-801 ura3-52 arf1-3 arf2Δ::LEU2*	[54]
DKARF1	MAT*α his3-Δ200 leu2-3,112 lys2-801 ura3-52 arf1-3 arf2Δ::LEU2 vps13Δ::natMX*	This work
CHC1-RFP	MAT**a** *his3Δ1 leu2Δ0 met15Δ0 ura3Δ0 CHC1-RFP::KanMX*	[80]
vps13Δ CHC1-RFP	MAT**a** *his3Δ1 leu2Δ0 met15Δ0 ura3Δ0 CHC1-RFP::KanMX vps13Δ::URA3*	This work
PTY44	MAT**a** *ura3-52 lys2 leu2-3,112 trp1Δ1*	[81]
JC8/55	MAT**a** *leu2 can1 kar1-1 rho0*	[82]
DFS160	MAT*α ade2-101 leu2 ura3-52 arg8Δ::URA3 kar1-1 rho0*	[83]
AJT5	BY4741 *trp1Δ-loxP-kanMX-loxP* [*rho+, TRP1*]	This work
AJT7	MAT**a** *arf1-3 arf2- trp1Δ-loxP-kanMX-loxP* [*rho+, TRP1*]	This work
AJT9	MAT**a** *arf1-3 arf2- vps13Δ::natMX trp1Δ-loxP-kanMX-loxP* [*rho+, TRP1*]	This work
AJT11	BY4741 *arf1Δ::natMX trp1Δ-loxP-kanMX-loxP* [*rho+, TRP1*]	This work
AJT13	BY4741 *vps13Δ::natMX trp1Δ-loxP-kanMX-loxP* [*rho+, TRP1*]	This work

Table S2. The plasmids used in this study.

Plasmid		Reference
pBluescript SKII		Agilent Technologies, Santa Clara, CA, USA
pUG35-VPS13	pUG35-VPS13-yEGFP3	[20]-
mt-RFP	pRS415-mt-RFP	[55]
pRS416-P_{GPD}	pRS416-P_{GPD}	[86]
MCP1	pRS416-P_{GPD}-MCP1	This work
VPS13-GFP	p415-P_{TEF1}-VPS13-GFP	[20]
VPS13ΔPH-GFP	p415-P_{GPD1}-VPS13ΔPH-GFP	This work
SEC7-RFP	pRS416-SEC7-mRFP	[88]
VPS13	pRS415-VPS13	[20]
VPS13A	p415-P_{TEF1}-VPS13A	[20]
pKF463	P_{tac}-GST•tag-Thrombin_c.s.-TEV_c.s.- 2×STOP	[87]
GST-yPH	pKF463-vps13-yPH(G3036-L3144)	This work
GST-hPH	pKF463-VPS13A-hPH(G3058-L3174)	This work
yPH-GFP	pRS415-P_{TEF1}-vps13-yPH(G3036-L3144)-GFP	This work
